# Sporadic and von Hippel–Lindau Related Hemangioblastomas of Brain and Spinal Cord: Multimodal Imaging for Intraoperative Strategy

**DOI:** 10.3390/cancers14225492

**Published:** 2022-11-09

**Authors:** Elio Mazzapicchi, Francesco Restelli, Jacopo Falco, Morgan Broggi, Laura Gatti, Pierpaolo Alongi, Laura Valentini, Paolo Ferroli, Ignazio G. Vetrano, Francesco DiMeco, Francesco Acerbi

**Affiliations:** 1 Neurosurgical Unit 2, Department of Neurosurgery, Fondazione IRCCS Istituto Neurologico Carlo Besta, 20133 Milan, Italy; 2 Neurobiology Laboratory, Fondazione IRCCS Istituto Neurologico Carlo Besta, 20133 Milan, Italy; 3 Nuclear Medicine Unit, ARNAS Ospedali Civico, Di Cristina e Benfratelli, 90127 Palermo, Italy; 4 Neurosurgical Unit 1, Department of Neurosurgery, Fondazione IRCCS Istituto Neurologico Carlo Besta, 20133 Milan, Italy; 5 Department of Biomedical Sciences for Health, Università di Milano, 20122 Milan, Italy; 6 Department of Oncology and Hemato-Oncology, Università di Milano, 20122 Milan, Italy; 7 Department of Neurological Surgery, Johns Hopkins Medical School, Baltimore, MD 21205, USA; 8 Experimental Microsurgical Laboratory, Department of Neurosurgery, Fondazione IRCCS Istituto Neurologico Carlo Besta, 20133 Milan, Italy

**Keywords:** CEUS, fluorescein, hemangioblastoma, ICG, indocyanine green, intraoperative imaging, ultrasounds, videoangiography, von Hippel–Lindau

## Abstract

**Simple Summary:**

Central Nervous System (CNS) hemangioblastomas (HBs) are rare and benign tumors that may be sporadic or hereditary, linked mainly to von Hippel–Lindau disease. Considering their slow-growing behavior and the possible risks related to surgical treatment, a careful, tailored approach should be adopted. This work aims to provide a systematic overview of the clinical appearance and neuroradiological characteristics of CNS HBs, along with a report of clinical–radiological data of a mono-institutional retrospective cohort, with particular emphasis on the possible role of intraoperative multimodal imaging to guide surgical strategy.

**Abstract:**

Hemangioblastomas (HBs) are rare, benign tumors often related to von Hippel–Lindau disease. They represent the most frequent primary cerebellar tumors in adults. Neurosurgical procedures aim to obtain a gross-total resection of tumor nodules, avoiding intra-postoperative hemorrhage. The introduction of new intraoperative imaging techniques has considerably changed surgical strategies in neuro-oncology. We present an overview of clinical and radiological data of a mono-institutional retrospective cohort, focusing on the role of intraoperative multimodal imaging in surgical strategy. From 2015 to 2021, we identified 64 (81%) cranial (42 cerebellar, 8 supratentorial, and 14 of the brainstem) HBs and 15 (19%) spinal (4 cervical and 11 dorsal) HBs in 79 patients. Intraoperatively, indocyanine green videoangiography with FLOW800 was used in 62 cases (52 cranial and 10 spinal), intraoperative ultrasound and contrast-enhanced ultrasounds in 22 cases (18 cranial and 4 spinal HBs), and fluorescein in 10 cases (in 6 cranial and 2 spinal cases used as SF-VA). Gross total resection was achieved in 100% of the cases (53 mural nodule removal and 26 complete resections of the solid tumor). No side effects were reported following the combination of these tools. Multimodal intraoperative techniques provide valuable and reliable information to identify the tumor and its vasculature, guiding a more precise and safer resection and reducing the risk of recurrence.

## 1. Introduction

Hemangioblastomas (HBs) are rare, highly vascularized, slow-growing benign tumors (WHO grade I), mainly composed of stromal and capillary vascular cells [1]. They account for about 2% of all intracranial neoplasms and 2–10% of primary spinal cord tumors, but HBs represent the most frequent primary cerebellar tumors in adults [2,3]. They can appear solid, solid–cystic, or mainly cystic with a small mural, vascularized node composed of stromal cells, and a dense capillary network [2,4]. Despite the great majority of HBs being sporadic (57–75%), they often represent a pathognomonic component of von Hippel–Lindau disease (VHL). Up to 72% of VHL patients develop one or more HBs in their life [2,5,6,7,8,9], determining the need for a strict follow-up. Sporadic HBs are most often located in the posterior fossa or spinal cord; supratentorial HBs are very rare. However, in recent series, a more frequent supratentorial localization of HBs in VHL disease has been reported [10,11]. Given their slow-growing behavior, the mean symptoms duration before the diagnosis can be up to several months. In VHL disease tumor cysts enlargement is much faster than in sporadic HBs leading to a prevalence of mass-effect symptoms derived from the cyst. These tumors often have periods of arrested growth, and many remain the same size for several years. These characteristics must be considered to propose an optimal management strategy. However, surgery can become necessary for patients primarily conservatively managed after the development/increase of mass effect [12]. The pivotal point of neurosurgical treatment is obtaining a gross-total resection of the nodule. To fenestrate or remove peritumoral cyst walls is unnecessary, considering that they often do not have a neoplastic activity and, after nodule resection, the cyst usually uniformly collapses [12]. In large and solid HBs, the potential for blood loss can be considerable, and these tumors can behave as arteriovenous malformations. In such cases, the first step is identifying and occluding the feeding arteries to avoid the risk of tumor congestion and intra- or postoperative hemorrhage [13].

In recent years, the introduction of new intraoperative imaging techniques has considerably changed surgical strategies in neuro-oncology, and these advancements also advanced the mainstays of HB surgery. For example, some studies have shown the role of intraoperative ultrasound (IOUS) in nodule detection [14,15], while contrast-enhanced ultrasound (iCEUS) analysis highlights the tumor and its vascular supply, also in deep-seated areas not directly visible on the brain or spinal cord surface [16]. Different fluorescent intraoperative dyes have been considered to help identify intramedullary HBs, thus reducing brain and spinal cord manipulation and the risks of postoperative worsening, increasing the extent of resection. Indocyanine green (ICG) with ICG videoangiography (ICG-VA) can visualize and identify vascular supplies of HBs [17,18], and the sodium salt of fluorescein (SF) has been used to localize the tumor and nodular areas [19]. However, these are mainly sporadic experiences; although all these techniques have been widely described for cerebral glioma surgery, their application has not been deeply characterized for HBs.

Hence, we present our experience with brain and spinal cord HBs, both sporadic and VHL-related, presenting the surgical and clinical results of a retrospective mono-institutional cohort, highlighting the possible impact that the implementation of a synergistic combination of different intraoperative imaging techniques may offer during surgical removal of such tumors.

## 2. Materials and Methods

**Figure 1:**
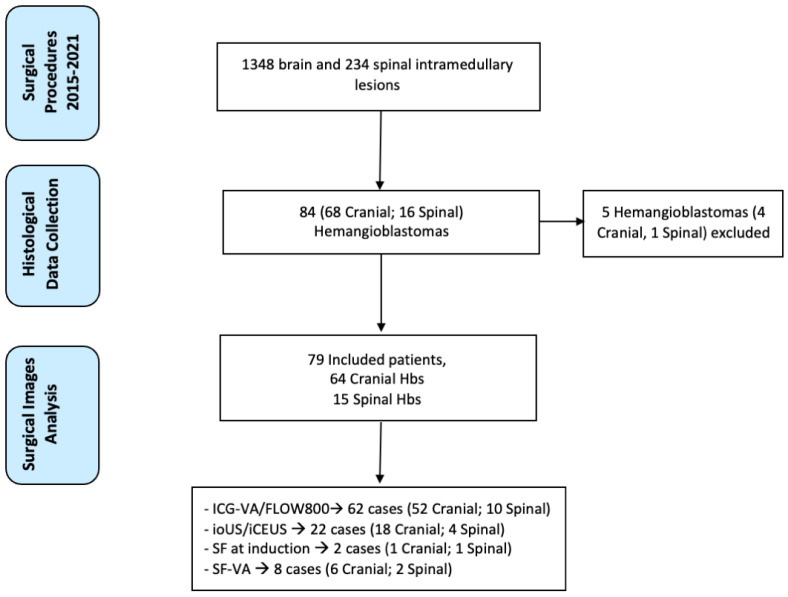
Graphical representation of the study depicting the flow of selection process.

A comprehensive retrospective analysis of surgical procedures involving HB resection at the Department of Neurosurgery of IRCCS Neurological Institute Carlo Besta between 2015 and 2021 was conducted (information was collected until January 2022). Five patients with incomplete surgical imaging data were excluded. For each patient, the following data were recorded and collected: age; sex; surgical approach; lesion localization and size; VHL mutation; the presence of hydrocephalus or syringomyelia; preoperative and postoperative MRI or CT exams; intraoperative use of ICG, SF, or iCEUS, as obtained by a complete review of the surgical videos and operating registers (Figure 1). When used, all patients gave their informed consent for undergoing ICG-VA, SF, or iCEUS administration. The local ethical committee approved the surgical database used for the retrospective analysis.

### 2.1. Surgical Protocol

A standard preoperative evaluation included blood tests and clinical assessments. All patients also underwent a preoperative MRI, including standard sequences for brain and spinal tumors. The general anesthesia was based on propofol intravenous administration under bispectral index monitoring. Tumor resection was performed with a standard technique based on tumor location, including the use of iCEUS, neuronavigation, photodynamic detection with fluorescence-guided technique, and neurophysiological monitoring if indicated. The specific approach and the surgical position strictly depended on the tumor size and location. For supratentorial HBs, the skin incision and a lesion-centered craniotomy after neuronavigation registration were planned. For posterior fossa/brainstem tumors, we usually adopted the semi-sitting position due to the possibility of having a more natural anatomical orientation and adequate cerebral venous decompression with easier CSF drainage [20]. For intramedullary HBs, surgery was performed with the patient in the prone position. The patient’s age and spine morphologic characteristics indicated laminectomy, laminotomy, or laminoplasty. Functional neurophysiological guidance was used when appropriate to preserve neurological function, especially if a myelotomy along the posterior sulcus was preoperatively planned [21]. Motor-evoked potentials (MEPs), sensory-evoked potentials (SEPs), and the D-wave, with established interpretation and warning criteria, were used for this purpose. SEP was also used to confirm the identification under a microscopic image of the midline sulcus, which the underlying tumor can asymmetrically distort.

### 2.2. Intraoperative Imaging Modalities

#### 2.2.1. IOUS and iCEUS

**Figure 2:**
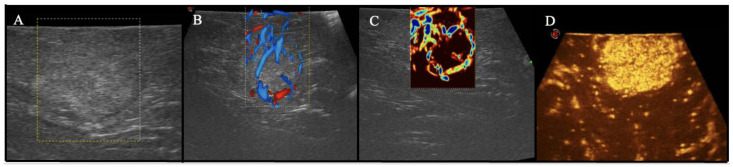
IOUS and iCEUS visualization of HBs: (**A**) standard B-mode examination showing HBs appear as hyperechoic lesions; (**B**) color Doppler used to evaluate the HBs’ perfusion pattern; (**C**) micro-V Doppler allows to obtain a micro-vascular perfusion analysis, depicting the surrounding vessels and the smaller capillaries inside the lesion; (**D**) iCEUS visualization showing a rapid and homogeneous enhancement of the nodular part.

After the craniotomy or laminotomy/laminectomy, patients were intraoperatively examined with transdural and direct sonography, using a last-generation US device (MyLab, Esaote, Italy) with a multifrequency linear US probe. The B-mode examination, in which ultrasounds are detected and visualized in a high-quality gray-scale image, was acquired to identify and measure the lesion on the major axes and its relationships with the surrounding structures. Tumors are described according to the difference of echogenicity compared to the surrounding structures, as hyper-, hypo-, or isoechoic (Figure 2A). Furthermore, color Doppler and advanced Doppler techniques (Micro-V) allow surgeons to visualize the perilesional vessels, create a map of the vascular tree, and study their hemodynamic pattern (Figure 2B,C). Ultrasound contrast agents (UCAs) are constituted by air or inert gas microbubbles encapsulated in a layer of proteins or polymers; they may increase the definition of tumor margins [22,23], better characterizing the vascular structures as well [24,25,26] (Figure 2D). UCAs can also permit, along with B-mode examination, to tailor the extent of myelotomy for intramedullary tumors [25].

#### 2.2.2. ICG Videoangiography with FLOW 800 Analysis

ICG, when requested, was injected intravenously by the anesthesiologist with a standard dose of 12.5 mg in a single bolus. In selected cases, based on surgeon necessity, is possible to perform multiple administration with a maximum daily dose of 5 mg/kg [19]. We used FLOW 800 software integrated into surgical microscope (Pentero or Kinevo, Carl Zeiss Meditec, Oberkochen, Germany) to perform the flow analysis. The software calculates and reconstructs color-coded maps based on fluorescence intensities and on time to half-maximal fluorescence in different vessels. This analysis provides parameters such as average intensity (shown in arbitrary intensity [AI] units), delay time, and the slope of the curve. In addition, the fluorescence flow could be further analyzed in brain parenchyma, by using freely definable regions of interest (ROIs), obtaining semi-quantitative flow information in that specific area.

#### 2.2.3. Sodium Fluorescein Protocol

The SF is a green, fluorescent synthetic water-soluble dye acting as a vascular fluorophore similar to the paramagnetic contrast agents in MRI. It is employed to improve the visualization and the extent of resection of different CNS and peripheral nerves tumors in which the blood–brain barrier (BBB) is disrupted. After intravenous administration during anesthesia induction, at the dose of 5 mg/kg [19], SF accumulates in CNS areas with altered vessel permeability due to BBB damage [27]. This dye needs a specific filter integrated with the surgical microscope, such as the YE560 filter of the Pentero or Kinevo microscope. SF can also be injected with a different modality, on demand, and with a lower dosage (75 mg) to highlight intracranial vessels in the form of intracranial videoangiography (SF-VA) [28,29]. With this modality, SF seems to allow for better recognition of feeding and draining vessels, acting similarly to ICG-VA but with a more detailed view compared to ICG-VA, as it also occurs for different brain and spinal tumors [30].

## 3. Results

### 3.1. Clinical Series

Among 1348 brain and 234 spinal intramedullary lesions, 79 HB patients were identified. Our study’s mean age of HB patients was 53 ± 12 years. The male/female ratio was 1:1.412 patients (15%) with multiple HBs diagnosed with VHL. Of the 79 cases analyzed, 64 (81%) were cranial HBs and 15 (19%) spinal HBs. Among the cranial HB cohort, 42 (65.6%) were cerebellar, 8 (12.5%) supratentorial, and 14 (21.8%) brainstem HBs. Looking at spinal HBs, 4 (26.6%) were located at the cervical level and 11 (73.4%) at the dorsal level. All patients underwent pre- and postoperative MRI, which demonstrated a total resection in 100% of the cases (53 (67%) with removal of the mural nodule and 26 (33%) with a complete resection of all the solid tumor). Hydrocephalus or syringomyelia was preoperatively present in 27 patients (34.1%). Of these, 16 presented with obstructive hydrocephalus and 11 with syringomyelia. In all cases of hydrocephalus, the lesion was removed, and a prophylactic external ventricular drain (EVD) was placed intraoperatively. Of these 16 cases, 9 EVDs were removed on the third postoperative day after evidence of radiological resolution of hydrocephalus, while 7 cases underwent a subsequent ventricle-peritoneal shunt. Syringomyelia showed progressive reduction or resolution, at the 3-moths follow-up, in 7 out of 11 patients. The patients’ demographics and clinical features are summarized in Table 1.

### 3.2. Use of Intraoperative Imaging Modalities during HB Resection

Regarding the use of intraoperative imaging techniques, ICG-VA with FLOW800 was used in 62 cases (52 cranial and 10 spinal), the IOUS and iCEUS in 22 cases (18 cranial and 4 spinal HBs), and SF was used in 10 cases. Specifically, SF was used in bolus on demand during surgical resection as SF-VA in 8 cases (6 cranial and 2 spinal), while only in two cases of spinal HBs we used the SF at the dose of 5 mg/kg at anesthesia induction, according to the FLUOCERTUM protocol [19]. ICG-VA was mainly used preoperatively to better identify the arterial feeders, the tumor nodule, and the arterialized draining veins visible on the surface and to better evaluate the flow alteration due to the HBs, thus improving surgical understanding of the lesion angioarchitecture and help in surgical strategy, reducing also the risk of intra-operative bleeding. In four cases of spinal HBs, the ICG-VA was also used to identify vascular afferents to the median raphe and improve the safety of myelotomy, as described by our group elsewhere [31]. In addition, it was also performed at the end of tumor to confirm total removal and the normalization of peritumoral vasculature after removal.

At the standard B-mode examination, HBs appeared as hyperechoic lesions, and the hypoechoic fluid cyst, when present, was well-visualizable too. Using iCEUS, all HBs examined appear as nodular hyperechoic masses, usually with a rapid and homogeneous enhancement of the nodular part, without cyst enhancement (Figure 2).

Spinal HBs appeared hyperechoic compared to the surrounding spinal cord, with a nodular, homogeneous aspect, and with perilesional cyst and macrocystic appearance or syringomyelic dilatation. In two tumors deeply embedded within the spinal cord, ultrasounds help correctly localize them, adapting the extent of myelotomy to the real tumor dimension. iCEUS was also used to identify the median raphe in spinal tumors in three cases: this was correctly identified in one case; in contrast, in two cases, iCEUS did not produce a clear identification image of the median raphe and was not helpful in guiding myelotomy.

**Figure 3:**
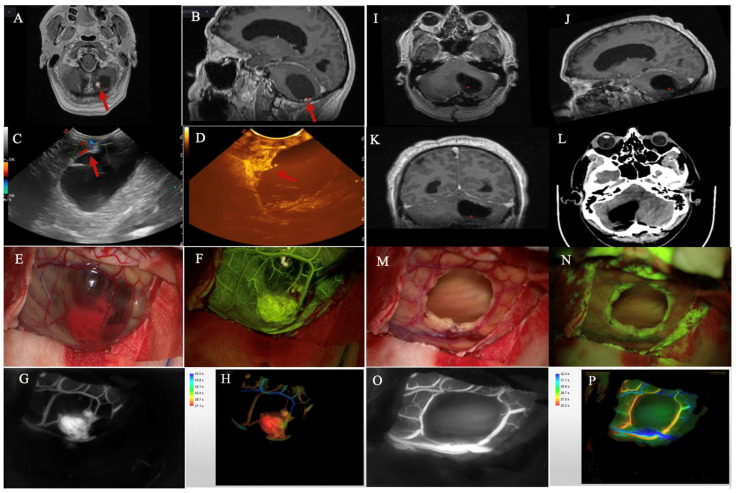
Cerebellar HB. (**A**,**B**) Preoperative axial and sagittal T1 after contrast medium administration MRI scans showing a right cerebellar HB, characterized by an intramural nodule (red arrow) and a cystic component. (**C**,**D**) IOUS and iCEUS images obtained after dural opening show the lesion’s cystic component, characterized by early enhancement of the intramural nodule (red arrow). (**E**) Intraoperative view of the lesion under white light. (**F**) Intraoperative view of the lesion after a bolus-type injection of SF to perform a SF-VA, which highlights the nodule and peritumoral cortical vessels. (**G**,**H**) Intraoperative vision after ICG-VA and FLOW800 analysis, evaluating perilesional hemodynamics. (**I**–**L**) Postoperative axial, sagittal, and coronal T1 after contrast administration MRI (**I**,**J**) and axial CT images (**L**) showing complete removal of the intramural nodule. (**M**,**N**) Intraoperative vision after lesion removal under white light and YELLOW560 filter. (**O**,**P**) Intraoperative control ICG-VA and FLOW800 showing preserved perilesional vessels with their quantitative blood flow evaluation.

SF-VA was able to show in all cases, in a similar way to ICG, arterial feeders, tumor nodules and draining veins (Figure 3).

**Figure 4:**
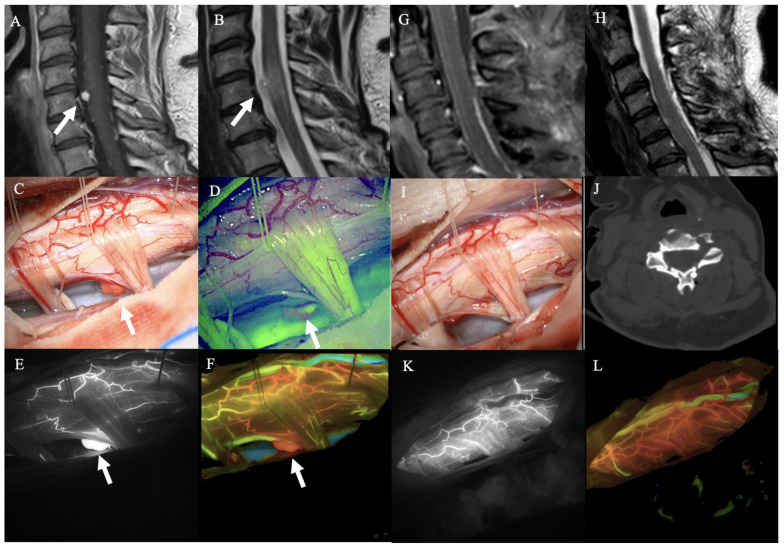
Cervical intramedullary HB. (**A**,**B**) Preoperative sagittal after contrast and T2-weighted sagittal MRI images showing a contrast-enhancing mural nodule in the anterior aspect of the cervical spinal cord, posteriorly to C5 body. (**C**) Intraoperative view of the lesion under white light illumination showing the mural nodule (white arrow) and its pial vessels communicating with it. (**D**) Under YELLOW560 filter, as expected, the nodule showed a low fluorescence, while the roots emerging from the spinal cord depicted spot of fluorescence too. (**E**,**F**) Intraoperative vision after ICG-VA and FLOW800 analysis with hemodynamics study of perilesional vessels. The mural nodule can be easily seen also under infrared visualization (white arrow). (**G**,**H**) Postoperative sagittal after contrast and T2-weighted sagittal MRI images showing the complete removal of the mural nodule, without surgical complications. (**I**) Intraoperative view after tumor resection, depicting the preservation of pial medullary vessels. (**J**) Postoperative CT scan, showing the laminoplasty. (**K**,**L**) Post-removal ICG-VA and FLOW800 images showing preserved perilesional vessels.

On the contrary, the use of SF at the moment of anesthesia induction, as described in FLUOCERTUM and subsequent studies [17,20], allowed only minimal fluorescence to be identified with the Y560 filter, without any clear advantage in tumor resection (Figure 4).

In our cohort, no side effects were reported following the administration of a single or multiple intraoperative contrast medium considered. Advantages and disadvantages of each technique are summarized in Table 2.

## 4. Discussion

Our series shows the feasibility of the contemporary administration of different contrast media for better and complete visualization of cranial and spinal HBs. The effectiveness of multimodal imaging for HB surgery is mainly due to the pathological characteristics of these benign but blood vessel-rich tumors. This allows the optimal use as contrast media with blood distribution and the lack of efficacy of metabolic agents. Surgery is indicated in case of mass effect due to cyst formation and growth or edema [12]. In all cases, the focus of surgical treatment is nodule resection; when an HB is associated with a contrast-enhanced cyst, to reduce the risk of recurrence, care is required to evaluate the possible neoplastic nature of the cyst’s wall [32]. In our series, we did not detect contrast-enhancing cysts’ walls. In such cases, cyst removal is unnecessary: after the nodule resection, the cyst usually collapses uniformly, as also occurred in all of our cases with cystic components. Another factor that must be considered is that most recurrences are due to incomplete resection, and regarding this aim, the role of intraoperative imaging techniques is of paramount importance.

The wide diffusion of fluorescent dyes in neuro-oncological surgery aims to increase the extent of resection safely. One of the most used fluorescent tracers in neurosurgery is 5-aminolevulinic acid (5-ALA). After oral administration, 5-ALA is metabolized into tumor cells [33]; however, despite its fundamental role in glioma surgery, the application of such dye in HBs is limited. As So Young Ji et al. reported, HBs do not seem to be fluorescent after 5-ALA administration [34]. On these bases, considering its putative mechanisms of action and the literature findings, we never administered 5-ALA as intraoperative fluorescence dye for HBS. ICG-VA and FLOW 800 have also been employed during the surgical removal of CNS tumors, with the specific aim of evaluating vascular abnormalities within the tumoral tissue, and also assessing the integrity of the regional vascular system after tumor resection [31,35]. Specifically, due to the intrinsic vascular abnormalities associated with HBs, the use of ICG-V to evaluate afferent vessels, draining veins and the tumor nodule would be ideal for such tumors. As a matter of fact, among the various intraoperative tools mentioned, the ICG-VA undoubtedly represents the most-used application in HB surgery. In 2014, Hojo et al. [36] presented one of the largest series reported in the literature, with 20 procedures performed. In this cohort, ICG-VA allowed for the identification of feeders and drainers; discrimination between transit feeders and adjacent non-feeding arteries; estimation of unexposed feeders; verification of complete resection and blood flow normalization in all cases. Regarding ICG-VA in spinal HBs, Hao and coauthors [37] reported their results in the application of ICG-VA in six cases of dorsal HBs, highlighting its utility in providing useful hemodynamic information during surgical procedures. In our 62 cases (52 cranial and 10 spinal HBs) with ICG-VA, this technique provided helpful information on discrimination between transit feeders and adjacent non-feeding arteries by carefully interpreting dynamic flow images under microscopic view. Estimating the tumor blood flow characteristics is also helpful in identifying hidden feeding arteries and draining veins that ICG cannot directly visualize.

Furthermore, we used ICG-VA, combined with intraoperative monitoring, to identify the posterior median sulcus when the tumor was not visible on the spinal cord surface and when the anatomy was distorted due to edema and congestion. In three HBs deeply embedded within the spinal cord, this technique allowed us an easier visual identification of the median sulcus. SF as a fluorescent tracer in neuro-oncology has shown significant advantages during high-grade gliomas, metastases, and other tumors’ resection [17,26,27,28,29], but experiences with HBs are very limited [30,38]. When used with the classic neurooncological protocol (5 mg/kg), SF does not seem to add a significant advantage in terms of better tumor visualization compared to the standard technique [19]. In the two HBs injected with SF at 5 mg/kg at anesthesia induction, the tumor bed showed only a slight fluorescence of the nodule. On the contrary, applying SF as a pure vascular fluorophore, the administration of fluorescein on demand upon surgeon request, with a lower dosage (75 mg), results in an intraoperative videoangiography [28]. With this modality, SF-VA recognizes feeding and draining vessels similarly to ICG-VA, as shown in the eight cases included in our series [30]. Moreover, unlike ICG or 5-ALA, the surgeon can continue the dissection under the YE560 light without switching between fluorescent and white light modes. Furthermore, the higher definition image of fluorescein angiography may be helpful in the detection of smaller perforators, especially in deep surgical fields with less illumination. In three out of eight cases, the SF-VA allowed us to identify small venous branches not evident on the ICG-VA.

The use of ultrasound during neurosurgical procedures has become more popular recently, and several studies have shown the role of this technique in tumor detection [14,15]. Intraoperative contrast-enhanced ultrasound (iCEUS) allowed to highlight neoplastic lesions and their vessels in deep compartments, not directly visible with ICG videoangiography [17,18,39]. In our series, at CEUS, HBs appeared as a nodular hyperechoic mass, usually with a rapid and homogeneous enhancement of the nodular part, while the cyst did not enhance. CEUS applications for HBs are, to date, really limited [16,17]. Della Pepa et al. [40] reported a single case in which iCEUS and color Doppler ultrasonography were used to perform intraoperative embolization of a posterior cranial fossa HB. Our group used CEUS for intraoperative identification of cervical spinal HBs, that were preoperatively diagnosed as gliomas [16]. Relying on our experience with iCEUS in brain tumor surgery, we used this method in 22 HBs cases. iCEUS is a valuable tool for visualizing neoplastic lesions better and obtaining additional information on their vascularity and perfusion. In one case of cerebellar HB, in which the preoperative diagnosis was pilocytic astrocytoma due to the contrast enhancement characteristics, the transdural CEUS depicted a straightforward and rapid enhancement of the nodule but not of the cyst’s wall. After dural opening, the tumor showed the classical macroscopic characteristic of an HB, and this finding was also confirmed after pathological analysis. This could be explained because of iCEUS providing dynamic and real-time perfusion imaging. Our findings helped approach the lesions and describe their perfusion patterns. iCEUS has proven to be a simple and relatively inexpensive technique representing a real-time dynamic procedure that can be performed during HB surgery.

## 5. Conclusions

The combination of SF, ICG-VA, and the intraoperative US with CEUS, can provide valuable and real-time information for identifying the tumor and its vasculature, thus guiding intraoperative decisions. Although the aforementioned intraoperative technologies are not always available, at the same time, in each center, their effectiveness and low cost, along with the information derived, provide essential information to guide a more precise and safer resection, potentially improving postoperative outcomes and reducing the risk of recurrence. All these techniques can be considered complementary and should be known by neurosurgical teams to optimize surgical resection and clinical outcomes.

## Figures and Tables

**Table 1:** Patients’ demographics and clinical features.

Patients’ Characteristics	Overall
N. Patients	79
Age, M ± SD	53 ± 12 years
Gender	
Male	32 (40.5%)
Female	47 (59.5%)
Hydrocephalus or Syringomyelia, *n* (%)	27 (34.1%)
VHL mutation	12 (15.1%)
Locations	
Cerebellum	42 (53.1%)
Brainstem	14 (17.7%)
Supratentorial	8 (10.1%)
Spinal cord	15 (18.9%)
Tumor Size (mm), M ± SD	24 ± 8

**Table 2:** Advantages and disadvantages of each single intraoperative imaging technique.

Technique	Dosage	Advantages	Disadvantages
iCEUS	2.4 mL	Completes and integrates standard B-mode and color Doppler imaging, providing dynamic and continuous real-time imaging and lesion characterization.	After tumor removal, bleedings or hemostatic agents could determine artifacts; need a learning curve mainly for anatomical orientation.
ICG-VA	12.5 mg	Noninvasive modality provides rapid information on the vascular flow dynamics allowing identification of tumor nodules.	No good visualization of deep lesions; necessary to wait at least 20 min before a new administration.
SF-VA	75 mg	More detailed identification of feeding and draining vessels. Good intraoperative visualization, no need to switch between fluorescent and white light mode during surgery.	Possible to do only a single administration per procedure.

## Data Availability

The data presented here are available on request from the corresponding author. The dataset will also be available at https://zenodo.org/ (accessed on 1 November 2022).
